# Mixture of *Salix* Genotypes Promotes Root Colonization With Dark Septate Endophytes and Changes P Cycling in the Mycorrhizosphere

**DOI:** 10.3389/fmicb.2018.01012

**Published:** 2018-05-18

**Authors:** Christel Baum, Katarzyna Hrynkiewicz, Sonia Szymańska, Nora Vitow, Stefanie Hoeber, Petra M. A. Fransson, Martin Weih

**Affiliations:** ^1^Soil Science, Faculty of Agricultural and Environmental Sciences, University of Rostock, Rostock, Germany; ^2^Department of Microbiology, Faculty of Biology and Environmental Protection, Nicolaus Copernicus University in Toruń, Toruń, Poland; ^3^Department of Crop Production Ecology, Swedish University of Agricultural Sciences, Uppsala, Sweden; ^4^Uppsala BioCenter, Department of Forest Mycology and Plant Pathology, Swedish University of Agricultural Sciences, Uppsala, Sweden

**Keywords:** arbuscular mycorrhizal fungi, ectomycorrhiza, soil enzymes, phosphorus, fine root density, short rotation coppice, willows

## Abstract

The roots of *Salix* spp. can be colonized by two types of mycorrhizal fungi (ectomycorrhizal and arbuscular) and furthermore by dark-septate endophytes. The fungal root colonization is affected by the plant genotype, soil properties and their interactions. However, the impact of host diversity accomplished by mixing different *Salix* genotypes within the site on root-associated fungi and P-mobilization in the field is not known. It can be hypothesized that mixing of genotypes with strong eco-physiological differences changes the diversity and abundance of root-associated fungi and P-mobilization in the mycorrhizosphere based on different root characteristics. To test this hypothesis, we have studied the mixture of two fundamentally eco-physiologically different *Salix* genotypes (*S. dasyclados* cv. ‘Loden’ and *S. schwerinii* × *S. viminalis* cv. ‘Tora’) compared to plots with pure genotypes in a randomized block design in a field experiment in Northern Germany. We assessed the abundance of mycorrhizal colonization, fungal diversity, fine root density in the soil and activities of hydrolytic enzymes involved in P-mobilization in the mycorrhizosphere in autumn and following spring after three vegetation periods. Mycorrhizal and endophytic diversity was low under all *Salix* treatments with *Laccaria tortilis* being the dominating ectomyorrhizal fungal species, and *Cadophora* and *Paraphaeosphaeria* spp. being the most common endophytic fungi. Interspecific root competition increased richness and root colonization by endophytic fungi (four taxa in the mixture vs. one found in the pure host genotype cultures) more than by ectomycorrhizal fungi and increased the activities of hydrolytic soil enzymes involved in the P-mineralization (acid phosphatase and β-glucosidase) in mixed stands. The data suggest selective promotion of endophytic root colonization and changed competition for nutrients by mixture of *Salix* genotypes.

## Introduction

Mycorrhizal fungi are central to soil fertility and can affect both crop productivity and cropping security (Rooney et al., 2009). Both arbuscular mycorrhizal (AM) and ectomycorrhizal (EM) fungi are known to increase the uptake of nutrients like phosphorus (P) and nitrogen (N) by the host plants, especially in infertile soils. However, their functions and benefits for the host plants might not be equivalent (Jones et al., 1998). For this reason, a change in the proportions of mycorrhizal colonization, or type of mycorrhizal association when the host plant can form dual mycorrhiza, might affect the mycorrhizal benefit of the host plant. The mycorrhizal colonization of plants can be affected by soil nutrient level and water content, site management as well as the diversity of the vegetation composition. An increased host plant diversity is known to influence the nutrient fluxes and the interactions with soil microorganisms (Courty et al., 2011). Endophytic fungi and mycorrhizal fungi can interact in their impact on the plant growth (Rillig et al., 2014). For example, AM fungi were revealed to be able to modulate the impact of endophytic fungi beneficially for the plant growth (Wężowicz et al., 2017).

Although the majority of higher plants form mycorrhizal associations, only a low number is dual mycorrhizal, forming both AM and EM associations (Smith and Read, 2008). *Salix* belongs to the dual mycorrhizal plant genera, and can also harbor diverse endophytic fungi, which may affect the hosts growth and ecophysiological traits (An et al., 2015). Although *Salix* spp. were mainly shown to be pre-dominantly EM (Püttsepp et al., 2004; Hrynkiewicz et al., 2010b), the opposite has also been shown with a dominance of AM colonization, e.g., in response to flooding (Lodge, 1989). The ability to change their mycorrhizal association in response to the environmental conditions makes them well-suitable model plants to investigate the changes in the mycorrhizosphere in response to a changed host diversity.

Fast growing *Salix* genotypes have been cultivated successfully in short rotation coppice (SRC) for a long time, mainly for biomass production for energy purposes in boreal climates (Weih, 2004). They are viewed as a sustainable source of biomass with a positive greenhouse gas balance due to their potential to fix and accumulate carbon (Cunniff et al., 2015). Furthermore, compared to annual systems grown on arable land, the management of perennial *Salix* stands in SRC leads to decreased mechanical disturbance of the soil and changed biochemical soil properties; along with changes in the abundance and diversity of soil organisms including mycorrhizal fungi (Baum et al., 2009a; Hrynkiewicz et al., 2010a; Fransson et al., 2013; Toju et al., 2013a; Goldmann et al., 2015). In addition, *Salix* genotype identity can significantly affect the soil enzyme activities at the same site (Baum and Hrynkiewicz, 2006), and *Salix* stands grown in a floodplain revealed higher activities of the soil enzyme β-glucosidase compared to perennial grassland (Zimmer et al., 2012). Changed soil enzyme activities are considered to be a direct expression of the soil community to metabolic requirements and available nutrients, and can therefore be used as indicators of soil functional diversity. Enzyme activities might be more indicative for ecosystem productivity and stability than the taxonomic diversity of soil microorganisms (Caldwell, 2005). Acid phosphatases and β-glucosidases are hydrolytic soil enzymes involved in P- and C-cycling of the soil and originate from plant roots and microorganisms. Mycorrhizal fungi can significantly increase the activities of these enzymes in the soil (Burke et al., 2011).

In SRC, *Salix* is usually planted with single genotypes, but this could possibly make them less resistant to pest organisms, diseases and abiotic stress. This is a major concern as a SRC must remain viable for up to 20 years to be profitable (Aravanopoulos et al., 1999). For this reason, an increased host plant diversity within *Salix* stands would be advantageous, but it will also change the below-ground plant–plant interactions within the stand. The soil ecological consequences of mixed *Salix* stands on former arable land are scarcely known so far (Hoeber et al., 2017).

We hypothesize that the mixture of genotypes with strong eco-physiological differences changes the mycorrhiza formation and activities in the mycorrhizosphere, based on different root characteristics of the genotypes involved. To test this hypothesis we have studied the mixture of two fundamental eco-physiological different *Salix* genotypes (*S. schwerinii* × *S. viminalis* cv. ‘Tora’ and *S. dasyclados* cv. ‘Loden’) grown in pure and mixed cultures in a field experiment in Northern Germany. Specifically, we expected (1) a higher diversity of root-associated fungi under mixed culture than in culture with the pure genotypes; and (2) changed fine root growth, abundance of root-associated fungi and enzymatic activities in the mycorrhizosphere under host genotype mixtures resulting from changed competitive conditions for the individual genotypes. Such insights into the overall structure of belowground plant–fungal associations will help to understand mechanisms that regulate the coexistence of eco-physiological diverse dual mycorrhizal plant species.

## Materials and Methods

### Study Site and Test Plants

The field site Rostock (54°04′12′′ N, 12°04′58′′ E) has been established in spring 2014 on former arable soil and is one out of three experimental field sites within the ECOLINK-*Salix* project (Hoeber et al., 2018), which is part of a global tree diversity network (TreeDivNet, Verheyen et al., 2016; Paquette et al., 2018). The plantation is a SRC stand with two willow genotypes [‘Tora,’ Svalöf-Weibull (SW) Cultivar No. 910007, *S. schwerinii* E. Wolf × *S. viminalis* L.; and ‘Loden’ SW 890129, *S. dasyclados*] grown in plots with one genotype being present (pure) and the combination (mixture) of the two. ‘Tora’ and ‘Loden’ in pure culture (‘Tora P’ and ‘Loden P’) were monocultures. In the mixture the two genotypes (‘Tora M’ and ‘Loden M’) were planted alternating one by one within the rows. Therefore, each genotype was directly associated with plants of the other genotype in the mixture.

Shoot biomass was harvested for the first time after 3 years of growth (in winter 2016/2017). The overall goal of the ECOLINK-*Salix* project is to assess the effects of genotype identity and diversity in willow SRC on various ecosystem functions. The experiment has a randomized block design, with three replicates (blocks, plot size: 92.16 m^2^). Planting density was c. 15600 plants ha^-1^. Each plot contains nine subplots (3.2 m × 3.2 m, 16 plants each).

The dominating soil type at the test site is a Stagnic Cambisol (FAO classification). The annual mean temperature is 8.5°C; the annual mean precipitation 592 mm. The texture class of the topsoil is predominantly loamy sand. General soil chemical properties (0–10 cm soil depth) were: pH (CaCl_2_) 6.2, C_org_ 6.89 g kg^-1^, N_total_ 0.86 g kg^-1^, C/N 8.01, P_total_ 0.11 g kg^-1^, and S_total_ 0.09 g kg^-1^.

### Soil and Fine Root Sample Collection

Soil and fine root sampling was done in November 2016 (at the end of the first rotation period after 3 years of growth) and in April 2017 (after the first shoot biomass harvest during the winter rest in February 2017). Nine soil cores (diameter 30 mm, depth 0–100 mm) per treatment were collected per genotype (2), sampling date (2), and planting layout (2: mono- [pure] and di-clonal [mix]) in the center of the plots for the determination of the fine root density, and of soil chemical and biochemical analyses (72 soil samples in total). The same number of samples and the same sampling design was used to collect the samples for the mycorrhizal analyses, but using 10 cm × 10 cm × 10 cm soil cubes taken with a sharp knife. The samples were collected from the uppermost 0–10 cm of soil, c. 20 cm from the stem base of willow plants and kept at 4°C until analyses. The majority of fine roots were found in 0–10 cm under diverse *Salix* species in SRC (Cunniff et al., 2015). For this reason the upper part of the topsoil seems to be most relevant for investigations of the mycorrhizosphere.

### Soil Properties

The soil analyses were done using air-dried < 2 mm soil. Determination of soil pH was performed electrometrically using a glass electrode in 0.01 M CaCl_2_ with a soil:solution ratio of 1:2.5. Total carbon (C_total_), total nitrogen (N_total_), and total sulfur (S_total_) contents of the soils were determined with a Vario EL elemental analyzer (Elementar Analysensysteme GmbH, Hanau, Germany). All C was organic C (C_org_) due to the acidic pH. The total phosphorus (P_total_) content was extracted from 0.5 g dry soil material by microwave-assisted digestion with aqua regia solution (3:1 hydrochloric acid–nitric acid) (Chen and Ma, 2001). The P concentrations in the digests were measured by inductively coupled plasma optical emission spectroscopy (ICP-OES) (Optima 8300, PerkinElmer LAS GmbH, Rodgau, Germany).

### Soil Enzyme Activities

The activities of acid phosphatases (EC 3.1.3.2) and β-glucosidases (EC 3.2.1.3) in the soil were determined colorimetrically according to Tabatabai (1982) and Eivazi and Tabatabai (1988). The enzyme activities were expressed as μg *p*-nitrophenol (pNP) g^-1^ soil h^-1^ released from the pre-given substrate solution (*p*-nitrophenyl-phosphate for acid phosphatases and *p*-nitrophenyl-β-D-glucosid for β-glucosidase) within 1 h of incubation.

### Fine Root Density

Soil cores with plant roots were soaked in tap water for 1 h in a bowl and then carefully separated with forceps and washed with the help of a sieve. Roots were dried at 65°C for 48 h and the dry weights were calculated per soil volume and m^2^ within the upper 0–10 cm soil depth.

### Fungal Colonization of Fine Roots

For analyses of AM and other endophytic colonization roots were rinsed and cut into 10 mm segments. The segments were cleared with 10% (w/v) KOH for 15 min at 90°C, bleached in 10% H_2_O_2_ for 1 h, acidified with 1% HCl (van der Heijden, 2001) and stained with 0.05% (w/v) chlorazol black E for 90 min at 80°C. The AM colonization was quantified microscopically using the intersection method of McGonigle et al. (1990), considering arbuscle formation as indicator of AM fungi vs. formation of sclerotia as indicator of dark septate endophytes (DSE). A minimum of 200 fine root intersections for AM colonization and a minimum of 200 fine root tips for DSE colonization were observed per plot.

For EM colonization and morphological characterization, 10 sub-samples of root fragments were randomly chosen on a grid for microscopical quantification of EM colonization. The numbers of living non-colonized root tips vs. obviously colonized EM root tips were counted using the method of Agerer (1991). In total c. 4800 root tips were scanned. A minimum of 200 root tips per genotype and treatment was investigated for each sampling date.

A minimum of two root tips per sub-sample was selected for subsequent molecular identification of the fungal partners. The morphological EM types were distinguished by macroscopical characteristics of the fungal mantle, such as color, surface appearance, presence of emanating hyphae and hyphal strands (Agerer, 1987–2002). Two to five root tips per sub-sample were separately frozen in Eppendorf-tubes and stored at -20°C for molecular analysis.

### Molecular and Phylogenetic Identification of Root Colonizing Fungi

The fungal taxa from the root samples were identified using analysis of DNA sequences of the internal transcribed spacer (ITS) region. DNA was isolated from root tips using the Plant & Fungi Purification Kit (EurX, Poland) according to the manufacturer’s instructions. The ITS region within the ribosomal RNA genes was amplified using the primer pair ITS1F and ITS4 (White et al., 1990; Gardes and Bruns, 1993). Amplification of the ITS region was performed in a 25 μl final volume containing 50 ng of DNA, 0.125 μl of each primer (100 pM/μl), 10 μl of Master Mix (Qiagen) and 13 μl of nuclease-free water. The amplification protocol was as follows: 5 min at 94°C, 40 cycles of 30 s at 94°C, 1 min at 50°C, and 1 min at 72°C, and a final step at 72°C for 5 min. The PCR products obtained were purified using GeneMATRIX PCR/DNA Clean-Up Purification Kit protocol (EurX, Poland) and sequenced using primers ITS1F and ITS4. Sequence editing was performed using Sequencher TW Version 5.1 (Gene Codes, Ann Arbor, MI, United States). BLAST searching with ITS-sequences was performed on the GenBank (Altschul et al., 1990). If the sequence of the fungus showed 98% identities over the whole length of the sequence (about 600 to 700 bp) with a known fungus, this fungus was assumed to be the fungal root colonizing taxa. Contigs of ITS sequences were edited with EditSeq and aligned using Clustal W (DNASTAR^®^). DNA sequences were submitted to GenBank and accession numbers are presented in **Table 1**.

**Table 1:** Molecular identification of fungal partners in fine roots of *Salix* genotypes ‘Tora’ and ‘Loden’ in pure (P) and mixed (M) growth design in 0–10 cm soil depth at the short rotation coppice Rostock in autumn 2016 and spring 2017.

Growth design	Clone	T bp	Closest BLAST match in GenBank (NCBI)^∗^ [Accession No.]/UNITE^∗∗^ or description of morphological features	Similarity %	Classified as [Accession No.]
**Ectomycorrhizal fungi**					
M	Loden	703	*Laccaria tortilis* voucher GMM7635 [KM067859]^∗^ *Laccaria tortilis* voucher DG05-14 [JQ888175]^∗^	702/703 (99%) 683/684 (99%)	*Laccaria tortilis* [MG076771]
M	Tora	721	*Laccaria tortilis voucher GMM7635 [KM067859]^∗^ Laccaria tortilis voucher DG05-14 [JQ888175]^∗^ Laccaria tortilis [KM067859]^∗∗^*	710/710 (100%) 684/684 (100%) 710/710 (100%)	*Laccaria tortilis* [MG076772]
**Endophytes**					
M	Loden	645	*Cadophora* sp. OTU097 AN-2016 (rhizosphere of *Plantago lanceolata*) [KU556579]^∗^* Leptodontidium orchidicola* strain ZT98 022 [AF486133]^∗^ Dark septate endophyte DS16b [AH008235]^∗^ *Cadophora* [KU556579]^∗∗^	641/642 (99%) 639/640 (99%) 640/643 (99%) 641/642 (99%)	*Cadophora* sp. [MG076773]
M	Loden	541ˆ	*Rhodotorula mucilaginosa* culture-collection CBS:329 *[KY104884]^∗^ Rhodotorula mucilaginosa [KY104887]^∗∗^*	474/511 (93%) 494/541 (91%)	*Rhodotorula mucilaginosa* [MG076774]
P	Tora	605	Uncultured fungus clone 99_NA1_P32_E12 isolated from the North American Arctic [KF297051]^∗^ (Timling et al., 2014) Uncultured endophytic fungus clone 374C-02 isolated from *Chorisodontium aciphyllum* (Antarctica) [KC457179]^∗^ (Zhang et al., 2013) *Eocronartium* sp. I12F-02262 isolated from *Sanionia uncinata* (Antarctica) [JX852332]^∗^ (Zhang et al., 2013) Pucciniomycetes [KF297051]^∗∗^	554/583 (95%) 502/530 (95%) 493/536 (92%) 554/583 (95%)	Pucciniomycotina [MG076775]
M	Tora	566	*Paraphaeosphaeria* sp. *sedF2 [KT265808]^∗^ Paraphaeosphaeria* sp. *OTU072 AN-2016 [KU556554]^∗^ Paraphaeosphaeria sporulosa [GU566257]^∗∗^*	556/561 (99%) 559/565 (99%) 557/561 (99%)	*Paraphaeosphaeria* sp. [MG076776]
M	Tora	590	*Pyrenochaeta* sp. strain P6099 [KT270292]^∗^ Fungal endophyte (*Holcus lanatus*) [FN392316]^∗^* Pyrenochaeta* sp. strain P2916 [KT270113]^∗^** Pleosporales [KF385302]^∗∗^	522/523 (99%) 518/518 (100%) 522/524 (99%) 570/587 (97%)	Pleosporales [MG076777]

*^∗^NCBI, ^∗∗^UNITE*.

### Statistical Analyses

The effect of the genotype-, the growth design (pure vs. mixed) and the interaction of both on biological and biochemical properties were analyzed by two-way ANOVA. The Detrended correspondence analysis (DCA) was used to disclose the effects of different host plant diversity (pure vs. mixed culture) on the root fungal colonization and soil enzyme activities in the mycorrhizosphere of *Salix.* Statistical analyses were computed using the software PAST (Hammer et al., 2001).

## Results

### Fine Root Biomass and Fungal Colonization

**FIGURE 1:**
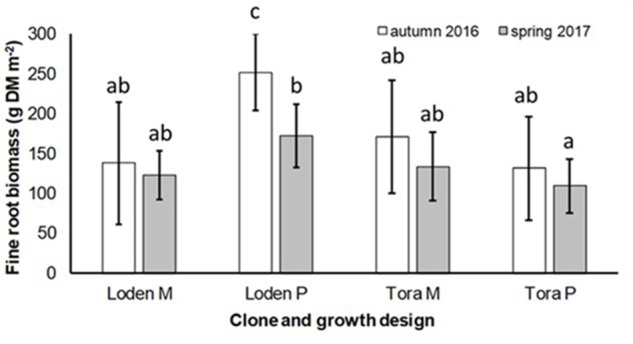
Fine root biomass (g DM per m^2^) under *Salix* genotypes ‘Tora’ and ‘Loden’ in pure (P) and mixed (M) growth in 0–10 cm soil depth at the short rotation coppice (SRC) Rostock in autumn 2016 and spring 2017 (means ± standard deviation, *n* = 9). Bars with different small letters (a, b, c) indicate significant differences among different treatments or sampling dates as defined by Duncan’s test (*p* < 0.05).

The fine root biomass was significantly affected by the interaction of genotype × growth design (pure vs. mixture) (**Table 2**). In pure culture ‘Loden P’ revealed higher fine root biomass than ‘Tora P,’ but under the mixture no significant differences between the two genotypes were observed (**Figure 1**). The means of the fine root biomass decreased in all treatments from autumn 2016 to spring 2017.

**Table 2:** Results of two-way analysis of variance (ANOVA) on the effect of the genotype, the growth design (with different host plant diversity; pure vs. mixture) and their interactions (genotype × growth design) on root and soil properties in the mycorrhizosphere of *Salix* from autumn 2016 (autumn) and spring 2017 (spring).

Parameter		Genotype		Growth design		Genotype × growth design	
		Autumn	Spring	Autumn	Spring	Autumn	Spring
Fine root biomass	*p*	0.142	0.056	0.207	0.352	**0.012**	**0.008**
	*F*	2.264	3.900	1.653	0.893	7.043	7.804
AM colonization	*p*	**<0.001**	**<0.001**	0.793	0.923	0.104	0.217
	*F*	130.400	9.500	0.070	0.009	2.796	1.587
ECM colonization	*p*	**<0.001**	**<0.001**	**0.005**	0.129	0.089	0.129
	*F*	96.340	84.250	8.898	2.418	3.064	2.418
DSE colonization	*p*	**0.009**	**0.012**	**<0.001**	**<0.001**	**0.022**	**<0.001**
	*F*	7.599	7.109	27.410	66.590	5.831	21.960
Activity of acid phosphatase	*p*	0.388	0.387	**<0.001**	**<0.001**	0.388	0.387
	*F*	0.766	0.763	25.110	25.110	0.766	0.763
Activity of β-glucosidase	*p*	**<0.001**	**<0.001**	**0.002**	**0.001**	**<0.001**	**<0.001**
	*F*	19.170	19.190	11.430	11.460	19.170	19.190

*p- Significance value (in bold are *p* < 0.05)*.

**FIGURE 2:**
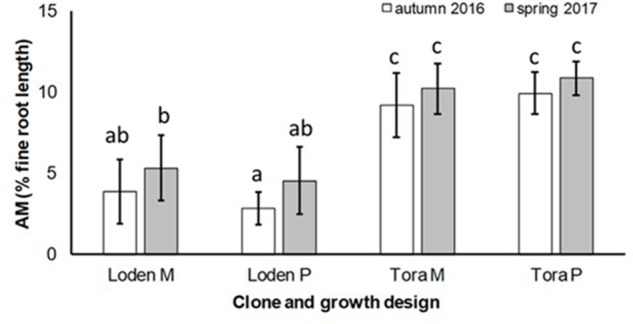
Arbuscular mycorrhizal colonization (% root length) and spore density (number of spores per 10 g DM soil) under *Salix* genotypes ‘Tora’ and ‘Loden’, in pure (P) and mixed (M) growth in 0–10 cm soil depth at the SRC Rostock in autumn 2016 and spring 2017 (means ± standard deviation, *n* = 9). Bars with different small letters (a, b, c) indicate significant differences among different treatments or sampling dates as defined by Duncan’s test (*p* < 0.05).

The AM colonization was relatively small (<12% of the total fine root length) in all treatments, but higher under ‘Tora M and P,’ than under ‘Loden M and P’ (**Figure 2**). The AM colonization of the fine roots was not significantly affected by the growth design (pure or mixed culture; **Table 2**). It was higher in spring than in autumn (**Figure 2**).

**FIGURE 3:**
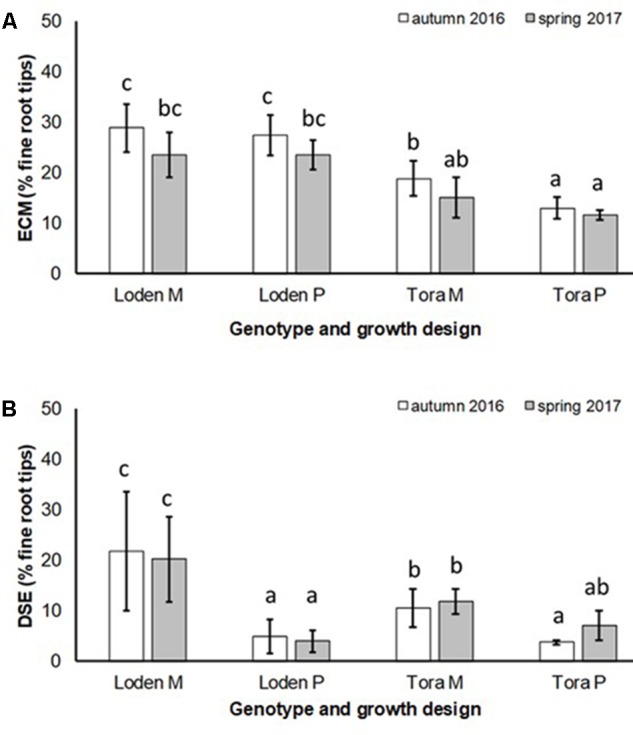
**(A)** Ectomycorrhizal colonization (ECM) and **(B)** dark septate endophytic colonization (DSE) of fine roots of *Salix* genotypes ‘Tora’ and ‘Loden in pure (P) and mixed (M) growth in 0–10 cm soil depth at the SRC Rostock in autumn 2016 and spring 2017 (means ± standard deviation, *n* = 9). Bars with different small letters (a, b, c) indicate significant differences among different treatments or sampling dates as defined by Duncan’s test (*p* < 0.05).

The EM colonization was significantly larger under ‘Loden M and P’ than under ‘Tora M and P,’ and significantly higher under the mixture in autumn 2016. In spring 2017 no significant impact of the growth design on the EM colonization was observed was (**Figure 3A** and **Table 2**). The EM colonization was in the mean slightly higher in autumn than in spring.

The DSE colonization was significantly larger in the mixture of genotypes (‘Loden M’ and ‘Tora M’) than in their pure cultures (‘Loden P’ and ‘Tora P’) (**Figure 3B**).

### Fungal Diversity on the Fine Roots

With more than 95% of the total EM colonization (data not shown), *Laccaria tortilis* dominated as the single fungal partner in EM formation in all plots and was isolated from the roots of both genotypes (**Table 1**). Sporocarps of *Laccaria tortilis* were observed in all plots during the autumn sampling 2016. Other rarely occurring EM morphotypes observed were morphologically–anatomically determined to belong to *Inocybe* and *Russula* spp. and were found under both clones.

One endophytic fungal taxa was identified from the genotype ‘Tora’ in pure culture (Pucciniomycotina) and four endophytic fungal taxa from the genotypes in mixed culture (*Cadophora* sp., *Paraphaeosphaeria* sp., *Rhodotorula mucilaginosa*, Pleosporales) (**Table 1** and Supplementary Figure 1).

### Soil Enzyme Activities

**FIGURE 4:**
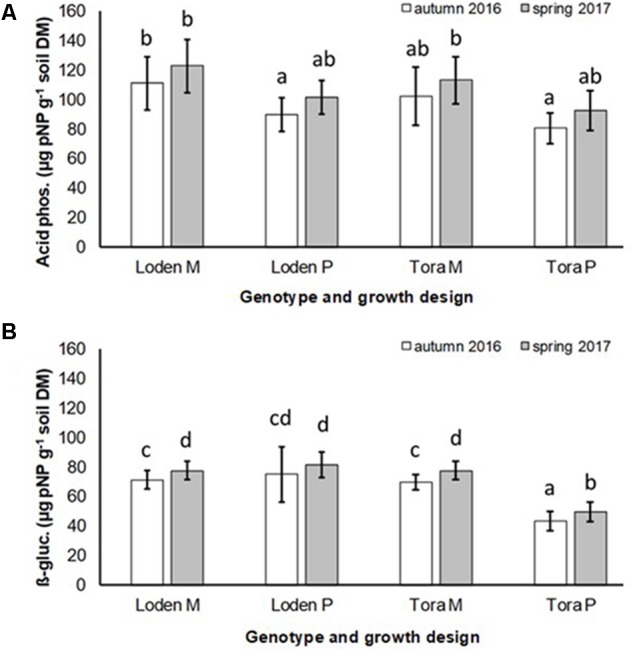
Soil enzyme activities of **(A)** acid phosphatase and **(B)** β-glucosidases of *Salix* genotypes ‘Tora’ and ‘Loden’ in pure (P) and mixed (M) growth in 0–10 cm soil depth at the SRC Rostock in autumn 2016 and spring 2017 (means ± standard deviation, *n* = 9). Bars with different small letters (a, b, c, d) indicate significant differences among different treatments or sampling dates as defined by Duncan’s test (*p* < 0.05).

The activity of acid phosphatase in the soil was significantly higher under the mixed growth design, but not significantly affected by the genotype and the interaction of genotype x growth design (**Figure 4A** and **Table 2**). The activities of β-glucosidase in the soil were higher under the genotype ‘Loden P’ in pure culture than under ‘Tora P’ in pure culture (**Figure 4B** and **Table 2**).

### Comparison Between Plots With Pure Host Genotypes and Genotype Mixtures

The mixed growth design of the genotypes increased significantly the colonization of fine roots by DSE, and the activities of acid phosphatases and β-glucosidases in the soil (**Table 2**).

**FIGURE 5:**
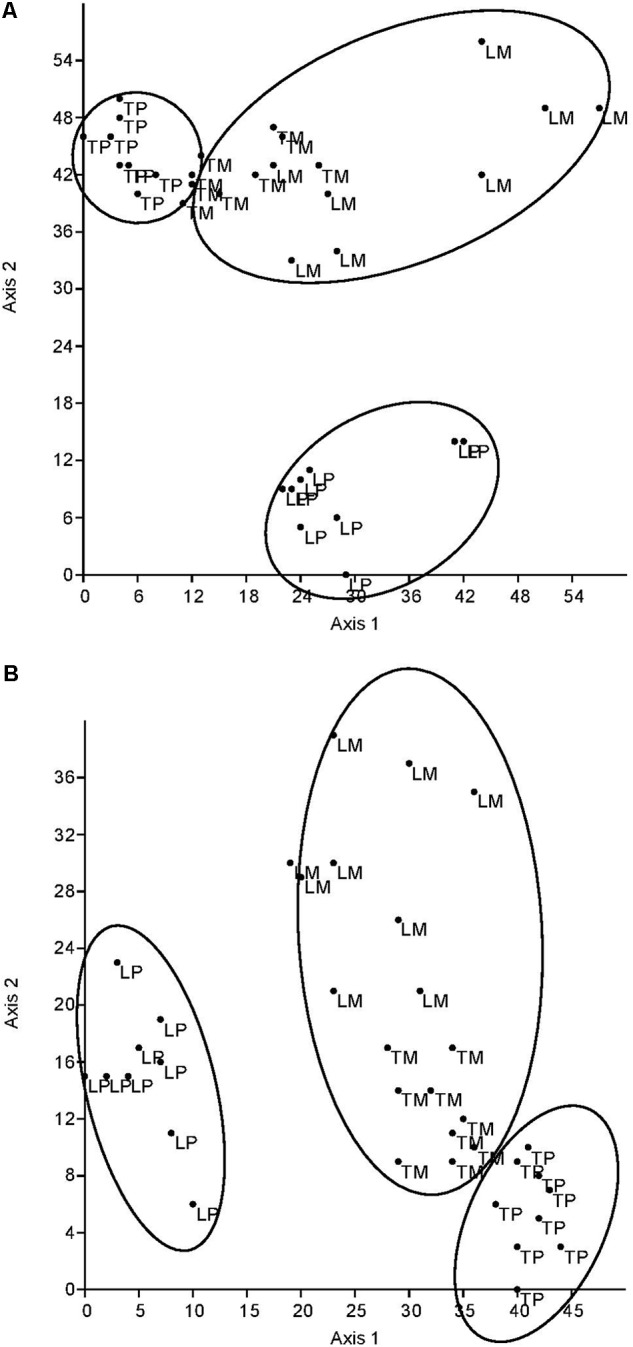
Detrended correspondence analysis (DCA) plot based on square root transformed, normalized data and Euclidean distance matrix. Comparison of the two planting designs [pure (P) and mixture (M)] of *Salix* [‘Loden’ (L) and ‘Tora’ (T)] by root and soil properties **(A)** in autumn 2016 (axis 1: Eigenvalue = 581.9, variance explained: 52.1%, axis 2: Eigenvalue = 281.9, variance explained: 25.2%) and **(B)** in spring 2017 (axis 1: Eigenvalue = 501.2, variance explained: 56.6%, axis 2: Eigenvalue = 230.8, variance explained: 26.1%).

The DCA of all fungal parameters and the two soil enzyme activities (acid phosphatase and β-glucosidase) clearly differentiated the genotypes (L for ‘Loden,’ T for ‘Tora’) and the host genotype mixture (M) from the two pure cultures (P) in autumn 2016 (**Figure 5A**) and spring 2017 (**Figure 5B**). The difference between the pure and the mixed culture was larger in spring 2017 than in autumn 2016, since the data differentiate the treatments along axis 2 (explaining about one quarter of the variation) in autumn, but along axis 1 in spring (explaining about half of the variation). The pure culture ‘Tora P’ was always closer to the mixture than the pure culture ‘Loden P.’

## Discussion

The present results confirm the significant impact of the *Salix* genotype on its phenotypical traits (Cunniff et al., 2015) including the mycorrhizal (see **Figures 2**, **3A**) and endophytic (see **Figure 3B**) fungal colonization and host-specific differences in the fungal root colonization within a plant community as described by Toju et al. (2013b). The soil properties of the test site indicated P-deficiency (see section “Study Site and Test Plants”), which increases the importance of mycorrhizal fungi for the P-supply of the host plants (Smith et al., 2011). Assuming that plant neighbors with a faster growth rate can increase the competition for plants with slower growth, in the present mixture ‘Tora M’ had an advantage over ‘Loden M.’ Both, decreased fine root biomass under ‘Loden M’ grown in a mixture (see **Figure 1**) and greater similarity of the root traits and soil enzyme activities of the mixture compared with pure cultures ‘Tora P’ or ‘Loden P’ support this assumption (see **Figure 5**). These results are in line with our hypothesis that fine root growth, fungal abundance and activities are changed under host genotype mixtures, caused by changed competitive conditions for the individual genotypes and thereby changed interactions between them. However, the genotype-effect on the mycorrhizal colonization exceeds the impact of the plant design (pure or mixture of genotypes) on the mycorrhizal abundance (see **Figures 2**, **3A** and **Table 2**).

We predicted that the increased diversity of host plants in the genotype mixture will increase the diversity of root associated fungi too, since aboveground and belowground diversity can be linked (De Deyn and Van der Putten, 2005). However, we found increased fungal diversity only in endophytic fungi, not in mycorrhizal ones. This increased diversity was associated with increased colonization of the roots with DSE. Generally, low mycorrhizal diversity on *Salix* found here by only one dominating EM fungal species (*Laccaria tortilis*; see **Table 1**) causes problems when interpreting the results. However, the low EM diversity is in line with the results on other genotypes of *Salix* (Püttsepp et al., 2004; Hrynkiewicz et al., 2012). It might be caused by the genotypic-specificity of this plant genus and additionally by the former arable site-conditions with lack of EM host plants. Therefore, we have focused rather on the ratios of root associations than on total mycorrhizal and endophytic diversity.

For the first time, we disclosed that increased host diversity of *Salix* genotypes can promote endophytic root colonization and increase soil enzyme activities involved in P-mobilization (see **Figures 2**–**4**). These results of a Continental SRC support the assumption of Pérez-Izquierdo et al. (2017) taken for Mediterranean forests, who highlighted the importance of structural shifts in fungal communities for their possible functional consequences. Functional consequences were revealed in an increased enzymatic P mobilization (see **Figure 4**) in the mycorrhizosphere with increased host plant diversity (mixture). These increased enzymatic activities can be caused directly by fungal impact, but also by the plant impact or most probably by a combination of both.

Dark septate endophytes were previously described to be the dominating root associated fungi under *S. caprea* on heavy metal contaminated sites (Likar and Regvar, 2013). In agreement with the results of these authors, we also found *Cadophora* spp. as a very common DSE in the roots of the two tested *Salix* genotypes at the non-contaminated arable site. This endophyte revealed plant growth promotion in heavy metal contaminated soils (Likar and Regvar, 2013). Also the second most common DSE in the present study (*Paraphaeosphaeria* sp., see **Table 1**), revealed plant growth promoting traits on another *Salix* sp. in heavy metal contaminated soil (An et al., 2015). The endophytic yeast *Rhodotorula mucilaginosa*, found under mixed *Salix* growth in the present study, was revealed to be plant growth promoting in *Populus* (Xin et al., 2009). However, from the present data no statement can be made on the growth response of *Salix* on the different fungal root colonization and the impact of DSE can vary from growth promotion to growth suppression of host plants (Aguilar-Trigueros and Rillig, 2016).

Increased colonization of the roots with DSE combined with consistent colonization with AM fungi in the mixture (see **Figures 1**, **3B**) were accompanied by increased soil enzyme activities involved in P-mobilization (see **Figure 4**). Della Monica et al. (2015) assumed, that DSE can be more effective to mobilize organic P, than AM fungi. However, in the present study increased enzyme activities might be stronger affected directly by the changed nutrient competition of the host plants in the mixture. Increased abundance of DSE without suppression of mycorrhiza formation supports that these fungal groups can interact beneficially in a joint host plant (Wężowicz et al., 2017). This might be caused by exudates of DSE, which can even stimulate the growth of mycorrhizal fungi (Scervino et al., 2009). Oppositely to the promotion of DSE in the mixture of different *Salix* genotypes, Becklin et al. (2012) revealed promoted of EM fungi on *Salix* in increased plant diversity by combination with herbaceous plants in an alpine meadow. However, these results have in common that host plant diversity changes the fungal root colonization of *Salix*.

The present data on dual mycorrhizal colonization of AM and EM fungi on *Salix* clearly confirmed the assumption of Lodge and Wentworth (1990), that these types of mycorrhizal fungi colonize roots with an antagonistic behavior. Interestingly, Chen et al. (2000) found that antagonistic behavior of AM and EM fungi was most severe when the EM fungus *Laccaria* was present, which was the dominating EM fungus in the present study. Low fungal diversity was observed also in the sporocarp production in other SRCs (Baum et al., 2002).

The slow-growing genotype ‘Loden’ seems to be rather EM dominated, whereas the fast-growing genotype ‘Tora’ seems to promote AM formation (see **Figures 2**, **3**). This might be affected by the different litter quality of these genotypes, since the litter quality affects mycorrhizal communities (Lambers et al., 1998). In a pot experiment, leaves of ‘Loden’ revealed higher phenolic concentrations in the later growth than ‘Tora’ under the same environmental conditions (Baum et al., 2009b). Leaf litter with higher phenolic concentrations was described to support EM formation, whereas low phenolic concentrations were found to promote AM colonization of the host plants (Lambers et al., 1998).

Interestingly, the colonization of the roots with DSE was stronger affected by the host plant diversity than by the genotype-specific differences, which was revealed by an increased colonization of the roots (see **Figure 3B**) combined with increased species richness (see **Table 1**) in the mixture. Increased stress tolerance of *Salix* spp. was previously linked to colonization by several DSE (An et al., 2015). However, root endophytes can also include potential pathogens and each plant species and combination with different fungal strains might respond differently in plant growth (Aguilar-Trigueros and Rillig, 2016). Furthermore, the colonization of roots with DSE is also affected by edaphic properties of the site (Xie et al., 2017). The impact of edaphic differences within the present test site was initially balanced between the treatments by the randomized plot design in the present study. However, the increased colonization by DSE might also lead back to changed nutrient mobilization caused by the changed root growth of at least one of the mixed genotypes (see **Figure 1**) and indicated by changed enzymatic P mobilization under the mixture (see **Figure 4**).

Based on our results, it can be concluded that increased genotype diversity in *Salix* cultivation can lead to changed root competition, fungal association and enzymatic P-mobilization in the mycorrhizosphere. Subsequent investigations should assess the generality of promotion of DSE and P-mobilization in increased host diversity.

## Author Contributions

MW conceived and designed the field experiment within the frame of ECOLINK-*Salix*. CB managed the field site Rostock, collected the samples, did soil and root analyses and statistical analyses and wrote the first draft of the manuscript; did all soil chemical and biochemical analyses and wrote part of Section “Materials and Methods.” NV and KH isolated the ectomycorrhizal fungi, created figures and tables and did statistical analyses. KH and SS identified the fungal species. NV analyzed the colonization density of mycorrhizal fungi and analyzed these parts of the results. SH and PF evaluated and discussed the mycorrhizal and endophytic results. All authors edited and revised the manuscript and approved the publication.

## Conflict of Interest Statement

The authors declare that the research was conducted in the absence of any commercial or financial relationships that could be construed as a potential conflict of interest.
