# Supplementary figures and images for: Mixture of Salix Genotypes Promotes Root Colonization With Dark Septate Endophytes and Changes P Cycling in the Mycorrhizosphere
Source: Front Microbiol. 2018 May 18;9:1012. doi: 10.3389/fmicb.2018.01012 (PMC5968087; doi:10.3389/fmicb.2018.01012)

Supplementary material 1: Phylogenetic tree of root-associated fungi on *Salix*

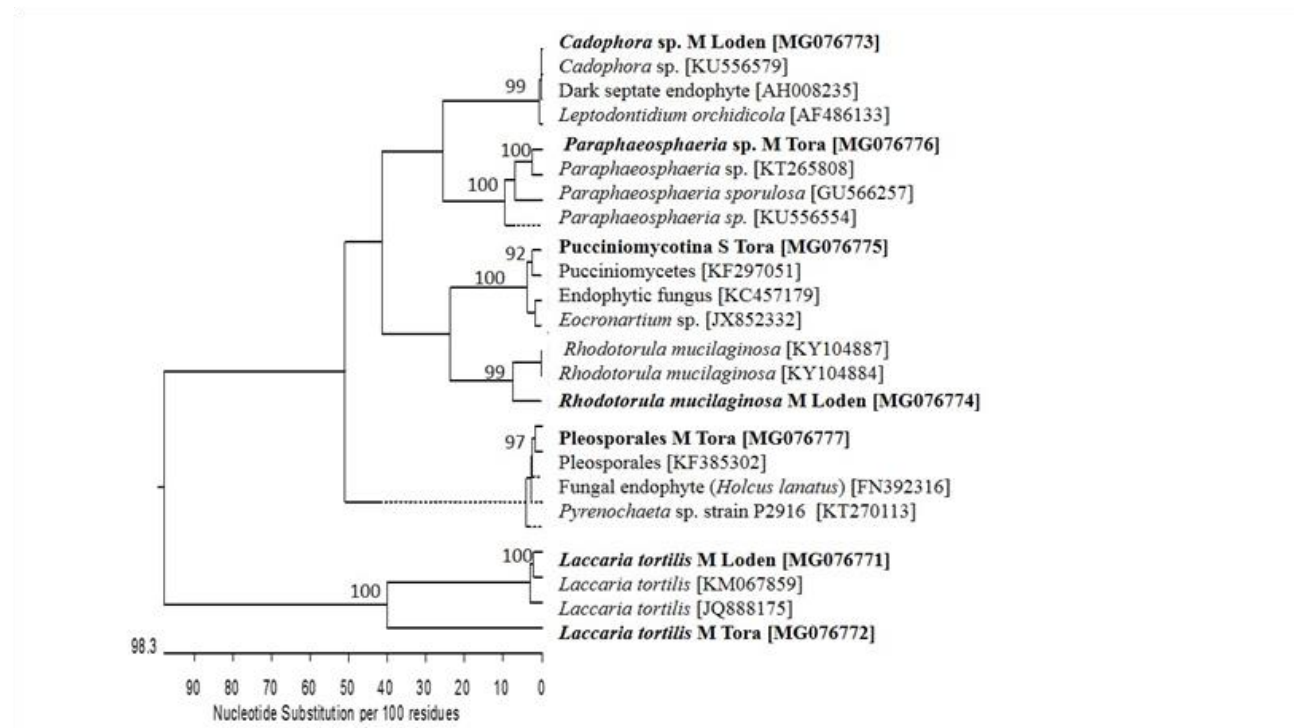

Supplement: Supplementary file 1 [file Image_1.pdf]
